# Enzyme-Assisted Release of Antioxidant Peptides from *Porphyra dioica* Conchocelis

**DOI:** 10.3390/antiox10020249

**Published:** 2021-02-06

**Authors:** Filipa B. Pimentel, Marlene Machado, Maria Cermeño, Thanyaporn Kleekayai, Susana Machado, Andreia M. Rego, Maria H. Abreu, Rita C. Alves, Maria Beatriz P. P. Oliveira, Richard J. FitzGerald

**Affiliations:** 1 LAQV, REQUIMTE, Department of Chemical Sciences, Faculty of Pharmacy, University of Porto, 4050-313 Porto, Portugal; marlenemachado753@gmail.com (M.M.); smachado@ff.up.pt (S.M.); beatoliv@ff.up.pt (M.B.P.P.O.); 2 Proteins and Peptides Research Group, Department of Biological Sciences, University of Limerick, V94 T9PX Limerick, Ireland; maria.cermeno@ul.ie (M.C.); thanyaporn.kleekayai@ul.ie (T.K.); dick.fitzgerald@ul.ie (R.J.F.); 3 ALGAplus—Produção e Comercialização de Algas e Seus Derivados, Lda., 3830-196 Ílhavo, Portugal; amrego@algaplus.pt (A.M.R.); htabreu@algaplus.pt (M.H.A.)

**Keywords:** *Porphyra dioica* conchocelis, amino acids, enzyme-assisted release, reactive oxygen species, antioxidant activity

## Abstract

The conchocelis life cycle stage of *P. dioica* represents an unexplored source of bioactive compounds. The aim of this study was to generate and characterise, for the first time, hydrolysates of conchocelis using a specific combination of proteases (Prolyve^®^ and Flavourzyme^®^). Hydrolysate molecular mass distribution and free amino acid contents were assessed, and the antioxidant activity was determined using a range of in vitro assays. The protein content and the total amino acid profiles of conchocelis were also studied. Conchocelis contained ~25% of protein (dry weight basis) and had a complete profile of essential amino acids. Direct sequential enzymatic treatment modified the profile of the generated compounds, increasing the amount of low molecular weight peptides (<1 kDa). There was a significant improvement in the antioxidant activity of the hydrolysates compared with the control (up to 2.5-fold), indicating their potential as a novel source of antioxidant ingredients.

## 1. Introduction

Currently, there is an increasing interest in natural antioxidants as an alternative to synthetic compounds, due to safety concerns and a worldwide trend of using natural food additives [1,2]. Indeed, natural antioxidants can shield the human body from free radicals or other reactive oxygen species and prevent or detain the development of many chronic diseases such as cancer, diabetes or atherosclerosis [2,3]. In addition, they can also be used in the food industry to inhibit, for example, lipid peroxidation, which is one of the main causes of food oxidation [3].

Reactive oxygen species (ROS) are highly reactive molecules that can be formed either by natural cell metabolism (endogenously) or by the action of different environmental factors, such as pollutants, ionising radiation and ultraviolet light (exogenously). Endogenous ROS have important physiological roles, such as protection of the cell against infection and regulation of intercellular signaling pathways [3]. However, when produced in excess, they can target membrane lipids, proteins, DNA and other bioactive macromolecules [4]. In foods, the oxidation process can contribute to the development of undesirable flavours and toxic substances, and consequently reduce consumer acceptance, thus becoming a concern for the food industry [4,5]. Therefore, the key approach is to use antioxidants or preservatives in order to prevent such oxidation in food products and to protect consumer health [6].

In contrast to natural antioxidants, synthetic antioxidants, such as butylated-hydroxytoluene (BHT) and butylated-hydroxyanisole (BHA), have better antioxidant activity and delay oxidation more efficiently [6]. However, these synthetic compounds are strictly regulated as they may have a negative impact on health [3]. In this way, a search for safe antioxidants of natural origin is necessary.

The antioxidant activity of food proteins derived from animal and plant sources has been widely reported [7,8]. Furthermore, the degradation of these proteins into peptides has demonstrated an increase in their antioxidant capacity due to their specific characteristics, e.g., amino acid sequence, hydrophobicity and molecular mass [3]. Antioxidant peptides can be generated from the original protein by controlled enzymatic hydrolysis, fermentation or released during digestion [9]. Enzymatic hydrolysis is the primary method of release of these molecules since it is considered a sustainable and rapid technique that can target specific amino acidic sequences. Moreover, enzymatic reactions are suitable in terms of food safety as they do not leave residues of organic solvents or other reagents that can be potentially harmful to human health [10]. As a result, the hydrolysates and peptides generated can be potentially used as agents in the prevention and management of certain diseases [11].

Seaweed is a rich source of antioxidants, which has received considerable attention from researchers, consumers and food industries [12]. Under severe environmental conditions, these macroalgae develop unique metabolic processes to survive, resulting in the synthesis of a wide range of secondary metabolites, some of which are antioxidant peptides [1].

The genus *Porphyra* is a red seaweed popularly known as “nori”. This is one of the most valuable macroalgal species in the world and most of its production is via aquaculture [13]. The life cycle of *Porphyra* involves two distinct phases. The gametophyte stage corresponds to the macroscopic blades while the microscopic filamentous sporophyte stage, known as the conchocelis stage, generally resides inside shells in natural habitats. *Porphyra* is characterised by a high protein content in the blade stage that can reach up to 45% in dry weight [5]. Although the conchocelis stage is still under-studied, Machado et al. [14] and Pimentel et al. [15] reported higher protein contents in the conchocelis compared to the blade stage.

The aim of this study was to ascertain the influence of enzymatic hydrolysis on the antioxidant properties of *P. dioica* conchocelis. Therefore, hydrolysates were prepared and characterised using a combination of proteolytic preparations (Prolyve^®^ and Flavourzyme). To our knowledge, this was the first time that this enzyme combination has been used to generate protein hydrolysates from *P. dioica* conchocelis. The molecular mass distribution, free amino acid content and antioxidant activity of the hydrolysates were studied, as well as the protein content and the total amino acid profiles of conchocelis.

## 2. Materials and Methods

### 2.1. Materials and Reagents

Flavourzyme^®^, a protease and exopeptidase preparation from *Aspergillus oryzae*, was supplied by Sigma (Dublin, Ireland) and Prolyve^®^ 1000, a protease from *Bacillus licheniformis*, was kindly provided by Lyven Enzymes Industrielles (Caen, France). HPLC-grade water and acetonitrile were from VWR International (Dublin, Ireland). Trinitrobenzene sulphonic (TNBS) acid solution was from Fisher Scientific (Dublin, Ireland). 2,2′-Azinobis-(3-ethylbenzothiazoline-6-sulfonate (ABTS), 2,2-diphenyl-1-picrylhydrazyl (DPPH), fluorescein, 2,2′-azobis-2-methyl-propanimidamide (AAPH), Trolox, amino acid standards, dihydrorhodamine 123 (DHR) and sodium hypochlorite solution (with 4% available chlorine) were obtained from Sigma (St. Louis, MO, USA). o-Phthalaldehyde/3-mercaptopropionic acid (OPA/3-MPA) and 9-fluorenylmethyl chloroformate (FMOC) were from Agilent Technologies (Palo Alto, CA, USA).

### 2.2. Algal Biomass

The algal biomass was provided by ALGAplus Ltd. (Aveiro, Portugal; production site: 40°36′43″ N, 8°40′43″ W). The *P. dioica* conchocelis phase was cultivated in an indoor nursery under controlled conditions. The collected biomass was washed with fresh water and was stored at −20 °C prior to freeze-drying (over 48 h at −80 °C and 0.015 mbar) using a Telstar Cryodos−80 (Terrassa, Barcelona, Spain). Dried samples were then stored in vacuum-sealed bags at room temperature (21.0 ± 0.2 °C) until use.

### 2.3. Determination of Nitrogenous Composition

A modified macro-Kjeldahl procedure [16] was used to determine total nitrogen (TN), non-protein nitrogen (NPN) and protein nitrogen (PN). Analyses were performed in triplicate (*n* = 3) and the results were expressed as % dry weight (dw). Protein content was estimated based on a nitrogen to protein conversion factor of 5.00 [17].

### 2.4. Hydrolysate Generation

Direct enzymatic hydrolysis of the algal biomass was sequentially performed using two food-grade and commercially available proteolytic preparations, Prolyve 1000^®^ and Flavourzyme^®^. Milled conchocelis were dispersed in deionised water (1:20, *w*/*v*) under gentle stirring for 30 min at room temperature. The suspension was then equilibrated at 50 °C and adjusted to pH 8.0 using 1.0 M NaOH. Incubation with Prolyve at an enzyme to substrate ratio (E:S) of 1% (*v*/*w* protein) was carried out under these conditions for 120 min. This hydrolysate was termed H-Prolyve. Hydrolysis was continued on further incubation following the addition of Flavourzyme at an E:S of 1% (*v*/*w* protein) at 50 °C and pH 8.0 for 120 min. This hydrolysate was termed H-ProFla. During the hydrolysis, a pH-STAT (Titrando 842, Tiamo 1.4 Metrohm, Dublin, Ireland) was used to maintain the reaction mixture at constant pH (8.0) using 0.5 M NaOH. Following each incubation step, the enzymes were inactivated by heating at 80 °C for 20 min in a water bath.

The hydrolysates and the control (sample treated under the same conditions without adding proteases) were centrifuged (11,950× *g*, 20 min, 10 °C; Hettich Universal 320R, Andreas Hettich GmbH & Co. KG, Tuttlingen, Germany) and the supernatants were freeze-dried (FreeZone 4.5 L, Labconco, Kansas City, MO, USA) and were subsequently stored in sealed bags in the dark at room temperature prior to further analyses.

### 2.5. Characterization of the Hydrolysates

The extent of hydrolysis was determined using the TNBS method as described by Le Maux et al. [18]. A leucine standard curve (0.0 to 2.0 mM, *R*^2^ = 0.9972) was used to determine the quantity of free amino groups released during the proteolytic reaction. Analyses were performed in triplicate and results were presented as mg N/g freeze-dried sample (FDS).

Gel permeation high performance liquid chromatography (GP-HPLC) analysis was used to determine the molecular mass distribution of the samples [19] while their peptide profiles were assessed by reversed-phase ultra-performance liquid chromatography (RP-UPLC) [20].

### 2.6. Total and Free Amino Acid Analysis

The total and free amino acids were determined as described by Machado et al. [21]. Briefly, total amino acids of conchocelis were quantified after submitting samples to chemical hydrolysis under alkaline (4 M KOH, 110 °C, 6 h) and acid (6 M HCl, 110 °C, 24 h) conditions. Free amino acids were extracted from the freeze-dried hydrolysate (H-Prolyve and H-ProFla) and the control (unhydrolysed) using solid-liquid extraction in deionised water (1.67% *w*/*v*), under repeated cycles of agitation (Multi RS-60, Biosan, Latvia) at room temperature for 45 min. Following centrifugation 17,000× *g*, for 10 min (Heraeus Fresco 17 centrifuge, Thermo Fisher Scientific, Osterode am Harz, Germany), the supernatants were collected and transferred into injection vials for amino acid analysis. L-Norvaline (2 mg/mL) was used as internal standard. Prior to HPLC analysis, samples were submitted to an automatic pre-column online derivatisation in an AS-4150 Autosampler (Jasco, Tokyo, Japan) using two derivatisation reagents (OPA/3-MPA and FMOC) as described by Machado et al. [21]. The individual amino acids were identified based on the retention time of known standards and quantified by the internal standard method. Extraction and determination of the total and free amino acid contents were performed in triplicate and the results presented as mg of amino acid/g FDS.

### 2.7. In Vitro Antioxidant Bioassays

A stock solution of each sample (hydrolysates and control) at 20 mg/mL was prepared in the appropriate buffer for each bioassay. Any undissolved material was removed by microcentrifugation (11,950× *g*, 5 min, 10 °C). Supernatants were collected and diluted as needed. The in vitro antioxidant activity was assessed using the following bioassays: oxygen radical absorbance capacity (ORAC) and ferric reducing activity power (FRAP) [22], HOCl [23], DPPH^•^ [24] and ABTS^•+^ [25] scavenging ability. For all the assays, Trolox was used as positive control and the results were expressed as μmol of Trolox equivalent (TE) per g of freeze-dried sample (μmol TE/g FDS) with the exception of HOCl expressed as IC_50_ value (half maximal inhibitory concentration).

### 2.8. Statistical Analysis

The results are presented as the mean ± standard deviation (SD) of at least three independent experiments. Data were analysed by one-way analysis of variance (ANOVA) at a significance level of *p* < 0.05, followed by multiple comparisons by Tukey’s post-hoc test. The statistical analysis was performed using GraphPad Prism 5 software (GraphPad, San Diego, CA, USA).

## 3. Results and Discussion

### 3.1. Protein Composition, Amino Acid Profile and Extent of Hydrolysis

The TN and NPN contents of conchocelis were 5.87 ± 0.01 and 0.78 ± 0.26% dw, respectively. The protein content (25.65 ± 0.35% dw) was estimated using the PN (5.13 ± 0.01% dw) and a conversion factor of 5.00, according to Angell et al. [17]. The E:S for subsequent proteolysis using Prolyve^®^ and Flavourzyme^®^ was estimated based on that conversion factor. A control of the conchocelis sample was incubated under the same conditions (temperature, pH and time) without proteases.

**Figure 1:**
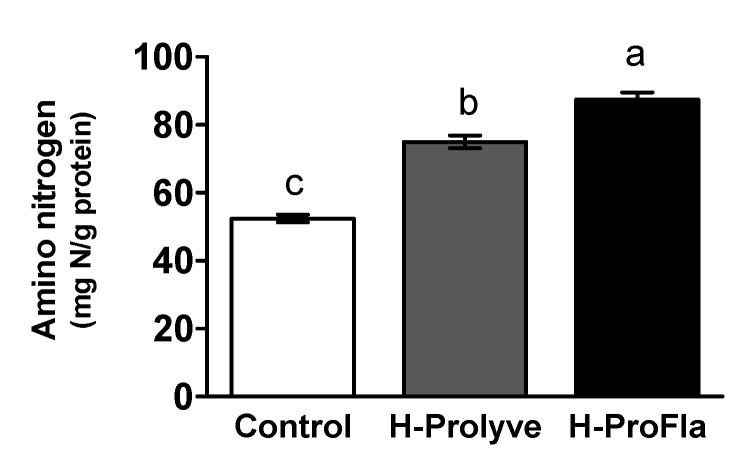
Amino nitrogen liberated from conchocelis after 2 h hydrolysis at 50 °C with Prolyve (H-Prolyve) and 4 h hydrolysis with Prolyve plus Flavourzyme (H-ProFla). Values represent mean ± SD (*n* = 3). Results expressed as mg of amino nitrogen per g of protein (mg N/g protein). Different letters denote significant differences at *p* < 0.05 for each sample.

As can be observed in Figure 1, the control sample presented 52.04 ± 1.13 mg N/g protein. A significant increase (*p* < 0.05) in the amino nitrogen content was observed between control and samples following 2 h hydrolysis with Prolyve and 4 h hydrolysis with Prolyve and Flavourzyme, i.e., 74.99 ± 1.88 and 87.38 ± 2.18 mg N/g protein for H-Prolyve and H-ProFla, respectively, indicative of proteolytic activity following incubation with the proteases.

The amino acid profiles of the samples are presented in Table 1. The most abundant amino acids in the conchocelis biomass were Asp > Glu > Ala > Arg > Leu > Gly > Lys > Val (28.74 ± 0.98, 27.55 ± 1.08, 24.98 ± 0.96, 18.84 ± 0.63, 18.42 ± 0.69, 18.04 ± 0.58, 17.91 ± 0.37 and 14.52 ± 0.51 mg/g FDS, respectively). Asn and Gln were not detected, as under the acid conditions used to determine the total amino acids, these are converted into Asp and Glu. The high levels of Asp and Glu found herein are consistent with the literature. Indeed, these results are in accordance with those found by Biancarosa et al. [26] for wild *P. dioica* blades collected in Northern Norway. In that study, the highest percentages were found for Ala, Asp, Glu, Gly Leu, Val and Lys. High levels of Asp, Glu, Ala and Leu have also been reported in the co-product generated following extraction of phycocolloids from *Porphyra columbina* [27].

The total amino acid (ΣAA) content was 245.0 ± 8.5 mg/g FDS. In fact, the true protein content can be estimated by the sum of amino acid residues [26]. Comparison of the true protein estimated from amino acids analysis (24.50 ± 0.85% dw) showed that there was no significant difference (*p* = 0.2916), in comparison to the value determined using the macro-Kjeldahl procedure (25.65 ± 0.35% dw). This demonstrated that a conversion factor of 5.00, as described by Angell et al. [17], was adequate to calculate the crude protein % (dw) of *P. dioica* conchocelis based on its PN content.

The proteolytic enzymes used during protein digestion generated hydrolysates containing free amino acids and peptides with different molecular mass [28]. Regarding the free amino acids profile of the hydrolysates H-Prolyve and H-ProFla, there was a significant increase (*p* < 0.05) in their amount, i.e., from 35.73 ± 0.79 to 48.36 ± 1.94 mg/g FDS after the complete enzymatic treatment. This suggests that this combination of proteases (Prolyve, a nonspecific microbial endoproteinase with subtilisin activity and Flavourzyme, a fungal enzyme complex with both endoproteinase and exopeptidase activities) promoted the generation of shorter peptides and the release of free amino acid residues in comparison to the hydrolysis with Prolyve alone.

The most abundant free amino acids in the hydrolysates H-Prolyve and H-ProFla were Ala, Glu, Arg, Leu, Val, Lys, Phe and Asp. The results showed that sequential hydrolysis using Prolyve and Flavourzyme converted approximately 20% of the total amino acids into free amino acid residues (Table 1). Aromatic amino acids (e.g., Tyr, Phe and Trp) are frequently reported to contribute to the antioxidant activity of peptides. Their ability to donate protons to electron-deficient radicals while maintaining their stability allows them to act as radical scavengers [29]. While sequential hydrolysis promoted the release of Tyr, Phe and Trp, the results indicate that ~76% of these residues were retained within the peptides generated by the enzymatic treatment. Therefore, it is possible that the peptides containing these amino acids were contributing to the hydrolysates antioxidant activity.

### 3.2. Peptide Profile and Molecular Mass Distribution

**Figure 2:**
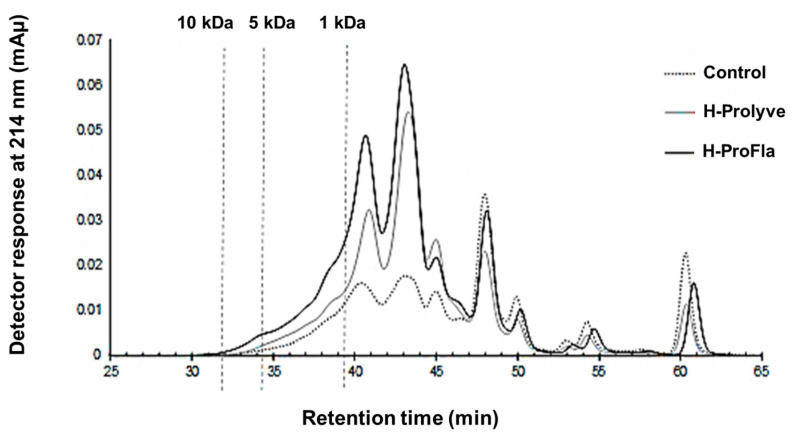
Gel permeation high-performance liquid chromatography profiles of *P. dioica* conchocelis following 2 h hydrolysis at 50 °C with Prolyve (H-Prolyve) and a total of 4 h hydrolysis with Prolyve plus Flavourzyme (H-ProFla). Dashed vertical lines indicate the retention times corresponding to proteins and peptides with masses of 10, 5 and 1 kDa.

Samples (control and hydrolysates) were submitted to GP-HPLC and RP-UPLC analysis to determine the contribution of hydrolysis to the protein/peptide profiles. Incubation of the conchocelis with proteases led to a decrease in molecular mass (Figure 2). Increasing the incubation time (from 2 to 4 h) and the addition of Flavourzyme led to significant reductions in peptide mass increasing the proportion of peptides <1 kDa.

**Figure 3:**
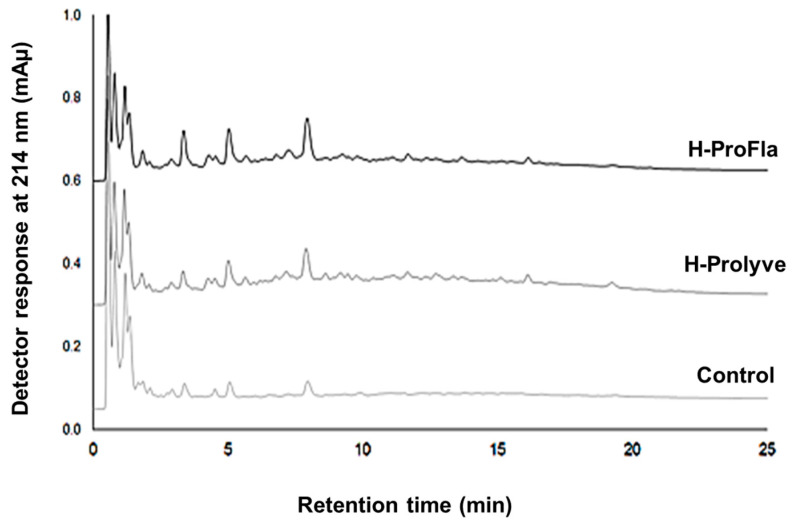
Reversed-phase ultra-performance liquid chromatography profiles of *P. dioica* conchocelis following 2 h hydrolysis at 50 °C with Prolyve (H-Prolyve) and a total of 4 h hydrolysis with Prolyve plus Flavourzyme (H-ProFla).

Incubation with the proteases also resulted in changes in the RP-UPLC profiles (Figure 3). Compared to control, a larger number of peaks were present following enzymatic treatment, especially in the hydrophilic region of the chromatograms (up to 10 min). Three high intensity peaks were present (retention times at ~3, ~5 and ~8 min) in the control, H-Prolyve and H-ProFla samples, the intensity of which intensity increased following enzymatic treatment. Some differences were observed in the peptide profiles, e.g., in the H-Prolyve sample low intensity peaks eluted up to 19 min, while in H-ProFla no peaks were evident after 16 min.

### 3.3. In Vitro Assessment of Antioxidant Activity

**Figure 4:**
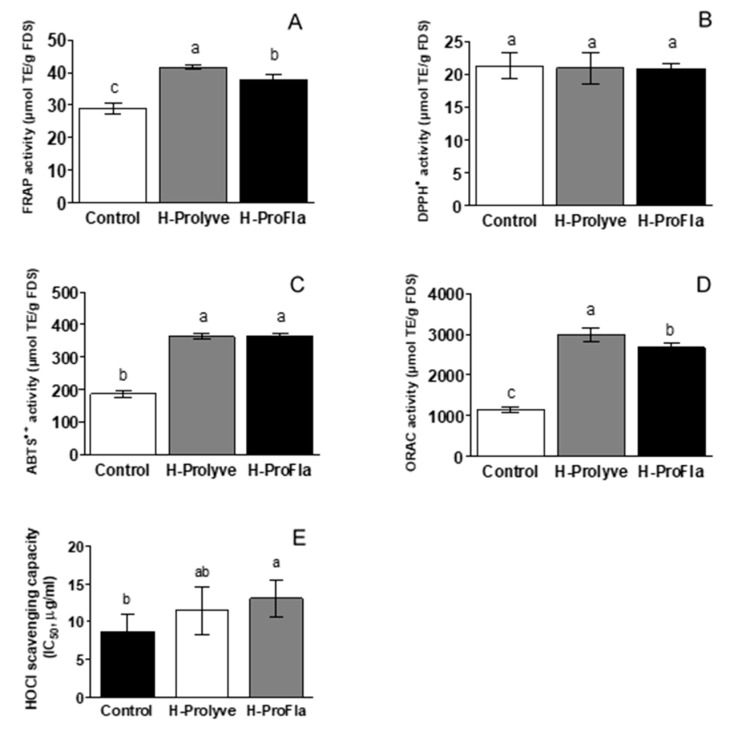
(**A**) Ferric reducing activity power (FRAP), (**B**) 2,2-Diphenyl-1-picrylhydrazyl (DPPH^•^) scavenging activity, (**C**) 2,2′-Azinobis-(3-ethylbenzothiazoline-6-sulfonate (ABTS^•+^) scavenging capacity, (**D**) Oxygen radical absorbance capacity (ORAC) and (**E**) Hypochlorous acid (HOCl) scavenging capacity of *P. dioica* conchocelis, following 2 h hydrolysis at 50 °C with Prolyve (H-Prolyve), and 4 h hydrolysis with Prolyve plus Flavourzyme (H-ProFla). Values represent mean ± SD (*n* = 3). Results expressed as μmol of Trolox equivalents (TE) per g of freeze-dried sample (μmol TE/g FDS). IC_50_ = inhibitory concentration, in vitro, to decrease by 50% the effect of the reactive species in the tested media (μg FDS/mL). Different letters denote significant differences at *p* < 0.05.

The associated metabolic mechanisms of food protein derived antioxidants are not fully understood. Therefore, different in vitro antioxidant assays are required to characterise antioxidative properties of food protein hydrolysates and peptides. Different methods were therefore selected based on complementary mechanisms of action in order to characterise the overall antioxidant profile of the samples. Indeed, some antioxidants can show activity in one assay and not in another, so it is always adequate to perform a wide range of in vitro antioxidant assays to study different mechanisms of action. In the case of DPPH^•^ and ABTS^•+^, both can be neutralised when receiving an H atom (radical quenching) and/or by electron transfer (direct reduction), while in the FRAP assay the TPTZ-Fe^3+^ complex is reduced to TPTZ-Fe^2+^ through an electron transfer mechanism by compounds with a redox potential below 0.7 V. In turn, in the ORAC method, fluorescein is protected from degradation by peroxyl radicals due to a H-transfer mechanism, and different compounds can also have the ability to scavenge HOCl through both ionic and radical mechanisms [30,31,32,33]. The results obtained in this study are summarised in Figure 4. Both hydrolysates presented significantly higher (*p* < 0.05) FRAP values compared to the control sample (28.86 ± 1.76, 41.62 ± 0.68 and 37.66 ± 1.85 μmol TE/g FDS, for control, H-Prolyve and H-ProFla, respectively) (Figure 4A). High FRAP values have been linked to the presence of sulphur-containing amino acids, e.g., Cys and Met [34]. The antioxidant activity of *P. dioica* blades was previously determined by our research group using the same protocol, i.e., direct hydrolysis of *P. dioica* blades using the same combination of enzymes, under the same conditions [5]. The FRAP values of the blade hydrolysates (29.59 ± 1.21 and 30.16 ± 0.99 μmol TE/g FDS, for H-Prolyve and H-ProFla, respectively) were lower than those found for conchocelis hydrolysates, indicating a higher reducing power of the latter. However, the sequential incubation of the conchocelis with Flavourzyme following Prolyve led to a decrease in the FRAP values (*p* < 0.05). Similar results have been reported for mushroom protein hydrolysates in which sequential proteolytic hydrolysis using Alcalase and Flavourzyme resulted in a decrease in FRAP values [35]. As the hydrolysis progresses, different amino acid side chain groups with electron-dense areas become more exposed. The presence of amino acid residues with bulky side chains (e.g., Ala, Val, Ile, Leu, Tyr, Phe, Trp, Pro and Met) in the conchocelis protein is noteworthy (Table 1). Peptides containing bulky hydrophobic side chains at their C-terminus are associated with high antioxidant activity. Additionally, the electronic and hydrogen-bonding properties, the location of the amino acids, along with the steric properties of the amino acid residues at the C- and N-terminal also contribute to the antioxidant activity of peptides [36]. The sequential incubation with the two proteolytic preparations may have released new peptides and free amino acids throughout the process. These compounds might act as an extra source of electrons and protons; hence, increasing the reduction potential of the hydrolysates. On the other hand, the larger molecular masses of the peptides in the H-Prolyve hydrolysate may be more favourable in relation to its reducing power.

The DPPH^•^ scavenging activity of the samples is depicted in Figure 4B. Despite presenting DPPH^•^ scavenging activity, no significant differences (*p* ≥ 0.05) were found between the conchocelis control and the hydrolysate samples (21.35 ± 2.04, 20.97 ± 2.35, and 20.88 ± 0.89 μmol TE/g FDS for control, H-Prolyve and H-ProFla, respectively). Likewise, the *P. dioica* blade samples presented DPPH^•^ scavenging activity, but no significant differences (*p* ≥ 0.05) were observed between the control sample and the corresponding hydrolysates (14.24 ± 0.77, 16.50± 0.56, 18.08 ± 1.44, for control, H-Prolyve and H-ProFla, respectively) [5]. When comparing the results of the two *P. dioica* life cycle stages, conchocelis samples present higher DPPH^•^ scavenging ability than the blade samples. The DPPH^•^ scavenging activity measures the ability of an antioxidant compound to donate electrons, which subsequently converts radicals into more stable species. While the results herein show that the test samples displayed DPPH^•^ scavenging ability, it would appear that proteolysis generated peptides with a similar ability to scavenge unstable radicals as the intact biomass when tested using the DPPH^•^ assay. A high proportion of Ala, Leu, Pro and aromatic amino acid residues (i.e., Trp, Phe, Tyr and His) has been associated with potent free radical scavenging activities [37].

Incubation with the proteases significantly increased (*p* < 0.05) the ABTS^•+^ scavenging capacity of both hydrolysates, compared to the control (186.9 ± 11.3, 363.6 ± 7.9 and 364.6 ± 6.3 μmol TE/g FDS for control, H-Prolyve and H-ProFla, respectively). Figure 4C shows that the radical scavenging ability of H-Prolyve and H-ProFla almost doubled following hydrolysis of the conchocelis. However, as already observed in the case of the DPPH^•^ assay, no significant differences were found between the ABTS^•+^ scavenging capacity of H-Prolyve and H-ProFla. The direct hydrolysis of the *P. dioica*-blade samples also increased the ABTS^•+^ scavenging capacity of the hydrolysates compared to the respective control (292.9 ± 10.6, 343.3 ± 11.5 vs. 121.8 ± 6.04 μmol TE/g FDS, for blade samples H-Prolyve, H-ProFla and control, respectively) [5]. Therefore, the conchocelis samples, including the control and the hydrolysates, displayed higher scavenging activity against the ABTS^•+^ radical when compared with the corresponding blade samples.

The conchocelis samples displayed high antioxidant responses in the ORAC assay. This is of interest as, within the in vitro assays used to assess the antioxidant activity of the samples, both the ORAC and the HOCl assays are considered as being biologically relevant. Enzymatic treatment of the conchocelis significantly increased (*p* < 0.05) the ORAC values of both hydrolysates compared to the control (1152 ± 71, 2995 ± 160 and 2676 ± 105 μmol TE/g FDS for control, H-Prolyve and H-ProFla, respectively). The ORAC values of H-Prolyve and H-ProFla were 2.5- and 2.3-fold higher than that of the control, respectively. Distinct differences in ORAC values were observed when comparing the *P. dioica* blade and conchocelis hydrolysate samples. In the case of the blade samples, the ORAC values significantly increased with the enzymatic treatment, i.e., from 609.9 ± 64.3 (control) to 2741 ± 239 and 3054 ± 333 μmol TE/g FDS (for H-Prolyve and H-ProFla, respectively) [5]. Interestingly, the ORAC value of the conchocelis control sample was almost twicethat of blade sample control (1152 ± 71 vs. 609.9 ± 64.3 μmol TE/g FDS, respectively), suggesting the presence of potent peroxyl radical neutralising compounds in the former. The presence of high levels of Pro and Tyr residues in peptides obtained from a hydrolysate of algal protein waste has been previously linked with its greater peroxyl radical scavenging capacity [38]. As shown in Table 1, these amino acids were also present in the conchocelis biomass (7.98 ± 0.48 and 9.17 ± 0.40 mg/g FDS for Pro and Tyr, respectively). While enzymatic treatment promoted some release of these amino acid residues, around 97% of Pro and 80% of Tyr was retained within the peptides generated. Despite increasing the antioxidant activity, incubation of *P. dioica* conchocelis with Flavourzyme after Prolyve led to a decrease in ORAC value. The increase in free amino acids in the H-ProFla sample (Table 1), probably associated with the exopeptidase activity in Flavourzyme may have contributed to the release of amino acids from the N- and C-terminal of peptides, thus generating peptides with lower peroxyl radical scavenging capacity.

All tested samples presented scavenging activity against HOCl with IC_50_ values in the µg/mL range (Figure 4E). It has been reported that sulphur-containing compounds efficiently prevent in vitro HOCl-induced deleterious effect [39]. Both the control sample, H-Prolyve and H-ProFla inhibited HOCl-induced oxidation of DHR-123 in a concentration-dependent manner (Supplementary Figure S1). However, the control sample presented the lowest IC_50_ value (8.69 ± 2.34 µg FDS/mL), suggesting that the greater the extent of hydrolysis, the lower the HOCl scavenging capacity of the hydrolysates (IC_50_ values: 10.29 ± 0.45 and 12.29 ± 0.54 for H-Prolyve and H-ProFla, respectively). These results may be explained by the fact that the reaction of HOCl with proteins can interfere with the enzymatic activity by modifying the reactive amino acid side-chains at or near the enzyme active site, leading to enzyme inhibition and loss of its structural function [40]. However, all samples presented significantly higher scavenging activity compared to Trolox (IC_50_ value: 116.5 µg/mL).

As already outlined, the antioxidant capacity of peptides is closely related to their structural characteristics, including their molecular mass, amino acid composition, sequence and hydrophobicity [36]. Peptides containing residues such as His, Trp and Tyr, can readily act as electron or proton donors, thus presenting enhanced peroxyl-scavenging capacity (ORAC assay) [41]. Nevertheless, some studies suggested that the amino acid sequence rather than the amino acid composition is the dominant factor influencing the bioactive properties of peptides [42,43]. However, the reactivity patterns of protein hydrolysates and peptides with radicals is complex and sometimes difficult to interpret, especially concerning the mechanisms involved. High antioxidant activity is often associated with short peptide sequences (3–6 residues) and peptides having low (<1 kDa) molecular mass [36,44]. Zhao et al. [45] isolated and identified ten new peptides from swim bladders of miiuy croaker with potent antioxidant activity, most of which contained 5–6 amino acid residues with molecular masses <1 kDa. The DPPH^•^ scavenging capacity of *P. columbina*-derived hydrolysates was associated with small molecular mass peptides (around 340 Da) and to free amino acids released during proteolysis, using a fungal protease concentrate and Flavourzyme [27]. A novel peptide (VECYGPNRPQF) generated from a *Chlorella vulgaris* co-product using pepsin was able to efficiently quench a variety of free radicals including DPPH^•^ and ABTS^•+^ along with superoxide, hydroxyl and peroxyl radicals. VECYGPNRPQF, with a molecular mass of 1309 Da, was considered a potent free radical scavenger, presenting higher activity than other compounds with recognised antioxidant activity, such as Trolox [38]. Peptides generated from a tuna by-product using Prolyve^®^ BS were isolated and assessed for their antioxidant activity. The novel peptides identified contained 5 to 8 amino acid residues having molecular masses ranging from 538.46 to 887.85 Da. The peptides exhibited good scavenging activities on hydroxyl, DPPH^•^ and superoxide radicals [29].

The present study allowed comparison of the differences in the in vitro antioxidant activity of the *P. dioica* blades [5] and conchocelis samples and their hydrolysates generated under similar hydrolytic conditions. The differences observed in the antioxidant activities may be more reflective of differences in the chemical composition of the seaweed at the different life cycle stages, rather than modifications promoted by the hydrolytic treatment. This is particularly relevant in the case of the ORAC values of the control samples in both studies. These differences may, in part, be related to the findings of Chan et al. [46]. The transcriptome analysis of two *Porphyra* speicies revealed differential expression of genes encoding for specific proteins (e.g., ribosomal proteins) which reflected specific regulatory processes associated with the different life phases. Therefore, enzymatic treatment of algal biomass from the same species at different life cycle stages, i.e., conchocelis and blades may have promote the release of peptides with different characteristics. Given that the starting material had distinctive compositions, it would be expected following enzymatic treatment that the resulting hydrolysates and/or peptides may also behave differently in the in vitro antioxidant assays.

Overall, hydrolysis increased (FRAP, ABTS^•+^, ORAC and HOCl scavenging activity) or maintained (DPPH^•^ scavenging assay) antioxidant activity of conchocelis compared with the unhydrolysed control sample. When comparing the results of conchocelis with blade samples submitted to the same protocol, major differences stand out regarding the ORAC assay where the conchocelis control sample presented higher antioxidant activity than blade control sample and, conversely, the blade H-ProFla sample presented higher antioxidant activity than the analogous conchocelis hydrolysate. This leads to the conclusion that the differences in reactivity of the samples against free radicals may be due to the chemical composition of the algal biomass that is inherent to the biosynthetic and biochemical processes of a particular life cycle stage. This may be of particular interest in developing a strategy to optimise the production of antioxidative compounds, i.e., from the biomass production step to the enzyme-assisted generation of hydrolysates and/or peptides. The present study is, to our knowledge, the first attempt to generate enzymatic hydrolysates from the conchocelis stage of *P. dioica* by direct hydrolysis of the algal biomass using a combination of proteases to enhance its antioxidant properties. Ultimately, our long-term goal is to develop seaweed-derived peptides and/or protein/peptide-rich ingredients with antioxidant activity.

## 4. Conclusions

Overall, proteolysis significantly improved the antioxidant activity of *P. dioica* conchocelis (particularly in the ORAC and ABTS^•+^ assays). This was attributed to the generation of low molecular weight peptides (<1 kDa) and/or amino acid residues with radical scavenging properties. This preliminary characterisation of *P. dioica* conchocelis biomass and its hydrolysates showing an improvement in their antioxidant properties following enzymatic treatment provides new evidence that conchocelis are a rich source of novel antioxidant ingredients, namely peptides. These can have potential to inhibit oxidation processes and thereby prolong food quality. In addition to their role in food preservation, they can also be useful in the generation of functional food ingredients/nutraceuticals.

Further characterisation of *P. dioica* conchocelis hydrolysates is still needed to identify those peptide sequences potentially responsible for the antioxidant activity and to clarify their potential physiological significance.

## Figures and Tables

**Table 1:** Total amino acid profile (mg/g freeze dried sample) of *P. dioica*-conchocelis and free amino acid profiles of *P. dioica*-conchocelis following 2 h hydrolysis at 50 °C with Prolyve (H-Prolyve) and a total of 4 h hydrolysis with Prolyve plus Flavourzyme (H-ProFla).

Amino Acids	Total Amino Acids	Free Amino Acids	
	*P. dioica*-Conchocelis	H-Prolyve	H-ProFla
Asp	28.74 ± 0.98	2.26 ± 0.06	2.12 ± 0.09
Glu	27.55 ± 1.08	8.48 ± 0.21	7.45 ± 0.29
Asn	n.d.	0.63 ± 0.01	1.19 ± 0.05
Gln	n.d.	0.53 ± 0.01	1.04 ± 0.04
Ala	24.98 ± 0.96	9.40 ± 0.22	9.40 ± 0.40
Arg	18.84 ± 0.63	1.86 ± 0.04	3.95 ± 0.17
Gly	18.04 ± 0.58	0.71 ± 0.01	1.12 ± 0.05
Ser	14.08 ± 0.50	0.91 ± 0.02	1.79 ± 0.07
Tyr	9.17 ± 0.40	0.88 ± 0.02	1.81 ± 0.07
Pro	7.98 ± 0.48	0.26 ± 0.02	0.23 ± 0.02
Hyp	0.05 ± 0.00	0.01 ± 0.00	0.01 ± 0.00
Phe	9.64 ± 0.36	1.44 ± 0.03	2.30 ± 0.08
His	6.99 ± 0.22	0.10 ± 0.01	0.33 ± 0.01
Ile	8.98 ± 0.32	0.75 ± 0.02	1.85 ± 0.08
Leu	18.42 ± 0.69	3.48 ± 0.09	5.49 ± 0.22
Lys	17.91 ± 0.37	1.00 ± 0.02	2.36 ± 0.09
Met	5.12 ± 0.20	0.23 ± 0.01	0.66 ± 0.07
Thr	12.70 ± 0.51	1.10 ± 0.02	1.71 ± 0.07
Trp	1.31 ± 0.13	0.44 ± 0.02	0.67 ± 0.05
Val	14.52 ± 0.51	1.26 ± 0.03	2.88 ± 0.12
∑AA	245.02 ± 8.47	35.73 ± 0.79	48.36 ± 1.94

Results presented as mean ± SD of 3 independent extractions. AA: amino acids; n.d.: not detected. Amino acids are represented using the 3-letter abbreviation.

## Data Availability

Not applicable.

## Supplementary Materials

The following are available online at https://www.mdpi.com/2076-3921/10/2/249/s1, Figure S1: Dose-response plots for the scavenging capacity of: (**A**) *P. dioica* conchocelis and its corresponding Prolyve (H-Prolyve), and Prolyve plus Flavourzyme (HProFla) hydrolysates; (**B**) trolox.
