# Supplementary material for: Enzyme-Assisted Release of Antioxidant Peptides from Porphyra dioica Conchocelis
Source: Antioxidants (Basel). 2021 Feb 6;10(2):249. doi: 10.3390/antiox10020249 (PMC7915985; doi:10.3390/antiox10020249)
Supplement: Supplementary file 1 [file antioxidants-10-00249-s001.pdf]

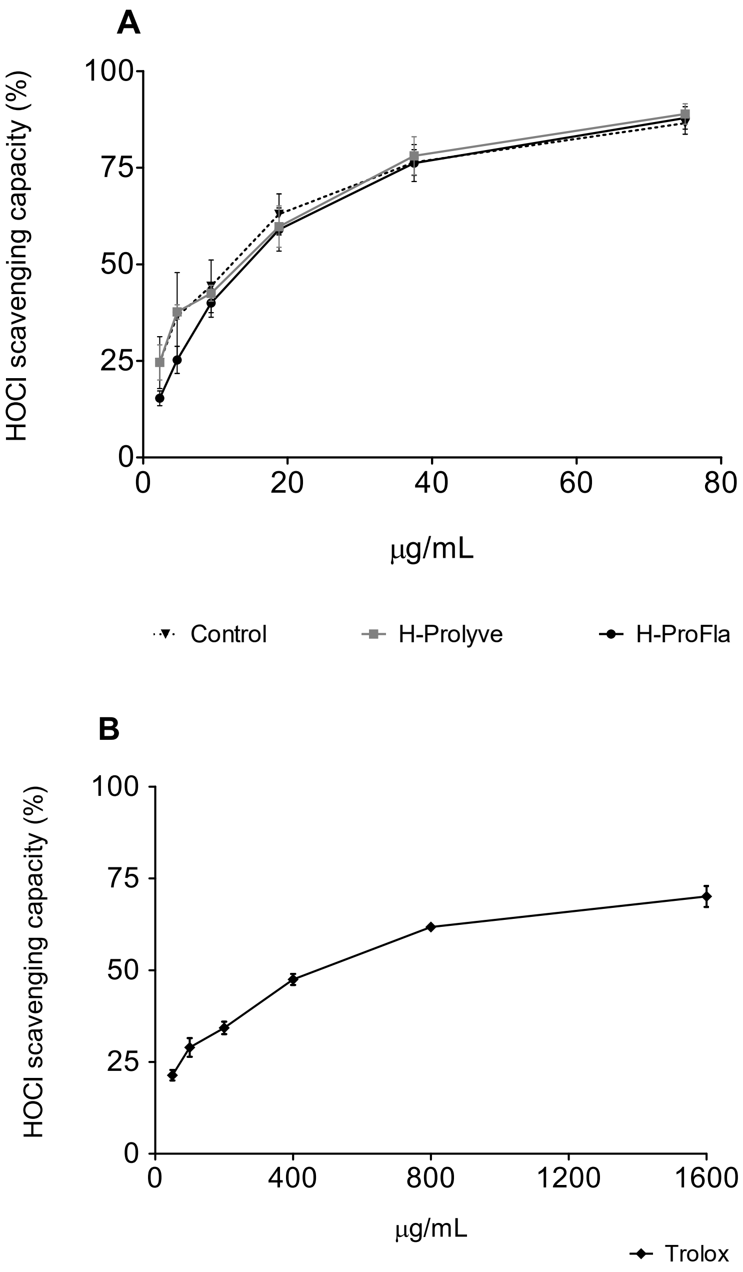

Figure S1. Dose-response plots for the scavenging capacity of: (**A**) *P. dioica* conchocelis and its corresponding Prolyve (H-Prolyve), and Prolyve plus Flavourzyme (H-ProFla) hydrolysates; (**B**) trolox. Error bars represent SD from at least 3 independent experiments, assayed at six different concentrations. The control sample corresponds to conchocelis incubated at 50 ºC for 4 h without enzymes.
